# Pan-Cancer Insights: A Study of Microbial Metabolite Receptors in Malignancy Dynamics

**DOI:** 10.3390/cancers16244178

**Published:** 2024-12-15

**Authors:** Nikolas Dovrolis, Michail Spathakis, Alexandra R. Collins, Varun Kumar Pandey, Muhammad Ikhtear Uddin, Donald D. Anderson, Tetiana Kaminska, Vasilis Paspaliaris, George Kolios

**Affiliations:** 1 Laboratory of Pharmacology, Department of Medicine, Democritus University of Thrace, 68100 Alexandroupolis, Greece; mspathak@med.duth.gr (M.S.); gkolios@med.duth.gr (G.K.); 2 Paspa Pharmaceuticals Pty Ltd., Hawthorn East, VIC 3145, Australia; ally@paspapharma.com (A.R.C.); varun@paspapharma.com (V.K.P.); muddin@paspapharma.com (M.I.U.); tkaminska@paspapharma.com (T.K.); bpaspa@tithonbiotech.com (V.P.); 3 BioGut Technologies Inc., Fort Worth, TX 76104, USA; danderson@biogut-tech.com; 4 Tithon Biotech, Inc., San Diego, CA 92127, USA

**Keywords:** microbiome, microbial metabolite receptors, cancer, pan-cancer, biomarkers, transcriptomics

## Abstract

Background/Objectives: The role of the gut microbiome in cancer biology has become an increasingly prominent area of research, particularly regarding the role of microbial metabolites and their receptors (MMRs). These metabolites, through the various gut–organ axes, have been proven to influence several pathogenetic mechanisms. This study conducted a comprehensive pan-cancer analysis of MMR transcriptomic profiles across twenty-three cancer types, exploring the mechanisms through which they can influence cancer development and progression. Methods: Utilizing both cancer cell lines from CCLE (Cancer Cell Line Encyclopedia) and human tumor samples from TCGA (The Cancer Gene Atlas), we analyzed 107 MMRs interacting with microbial metabolites such as short-chain fatty acids, bile acids, indole derivatives, and others while studying their interactions with key known cancer genes. Results: Our results revealed that certain MMRs, such as *GPR84* and serotonin receptors, are consistently upregulated in various malignancies, while others, like *ADRA1A*, are frequently downregulated, suggesting diverse roles in cancer pathophysiology. Furthermore, we identified significant correlations between MMR expression and cancer hallmark genes and pathways, including immune evasion, proliferation, and metastasis. Conclusions: These findings suggest that the interactions between microbial metabolites and MMRs may serve as potential biomarkers for cancer diagnosis, prognosis, and therapy, highlighting their therapeutic potential. This study underscores the significance of the microbiota–cancer axis and provides novel insights into microbiome-based strategies for cancer treatment.

## Simple Summary

Research increasingly shows that bacteria, the largest part of the gut microbiome, may play a significant role in cancer. These microorganisms produce metabolites that can travel through the body and interact with host cells via microbial metabolite receptors, potentially affecting cancer development. This study investigates the involvement of these receptors in human cells across twenty-three types of cancer. By analyzing data from both cancer cell lines and human tumor samples, we examined how these interactions may impact key cancer-related processes, such as immune response, tumor growth, and spread. Notably, we identified several receptors that are consistently altered in cancer, which might serve as helpful biomarkers for diagnosis or treatment. This research highlights the potential of targeting the gut microbiome in cancer therapy and provides valuable insights for developing new cancer treatments based on microbiome interactions.

## 1. Introduction

The study of the human microbiome has emerged as a pivotal area of research in contemporary biology and medicine. The microbiome, encompassing the trillions of microorganisms residing in the human body and even inside tumors [1], plays a crucial role in maintaining physiological homeostasis and modulating disease processes. These microorganisms, including bacteria, viruses, fungi, and archaea, engage in complex interactions with their host, influencing various aspects of health and disease. Among the many facets of microbiome research, the interplay between microbial metabolites and their respective receptors in the context of cancer biology has garnered significant attention.

Microbial metabolites are small molecules produced by the gut microbiota during the fermentation of dietary components and through other metabolic processes. These metabolites, such as short-chain fatty acids (SCFAs), bile acids, and indole derivatives, can profoundly impact host physiology by interacting with specific host microbial metabolite receptors (MMR). However, MMRs do not exhibit absolute specificity for microbial-derived ligands and can also be modulated by a variety of factors, including host-derived molecules, dietary components, and even cancer-associated metabolites, such as the SCFA lactate. This lack of absolute specificity allows MMRs to function as key mediators in complex signaling networks at the host–microbe interface [2]. These interactions can modulate immune responses, inflammation, and even cellular proliferation and differentiation, all critical factors in cancer development and progression. The MMR-mediated mechanisms, through which microbial metabolites exert their effects, are diverse. G-protein coupled receptors (GPCRs), nuclear receptors, and ligand-gated ion channels are among the key receptor families that respond to microbial metabolites [3].

In the context of cancer biology, the interactions between microbial metabolites and their receptors can have both tumor-promoting and tumor-suppressing effects, impacting cancer pathophysiology, prognosis, and treatment [4]. Gut microbial metabolites have displayed direct and indirect anticancer activities through different molecular mechanisms [5,6]. For example, Butyrate, a well-studied SCFA, has been shown to induce apoptosis and inhibit proliferation in colorectal cancer cells through its action on histone deacetylases (HDACs) and GPCRs [7,8]. Conversely, certain secondary bile acids, modified by the intestinal microbiota, have been implicated in promoting liver and colorectal carcinogenesis by inducing DNA damage and modulating cellular signaling pathways [9]. This knowledge enhances our fundamental understanding of cancer pathogenesis and opens new avenues for therapeutic intervention. Microbial metabolites seem to influence the efficacy of anticancer therapies; while targeting specific microbial metabolites or their receptors, it may be possible to develop novel strategies for cancer prevention and treatment [10,11]. Furthermore, the role of the diet in shaping the microbiome and its metabolic outputs is an area of growing interest. Dietary components such as fiber, fats, and polyphenols can influence the composition and activity of the gut microbiota, but the microbiota can also modify the dietary components and consequently the metabolites [12,13]. Therefore, modulation of the production of diet-derived microbial metabolites and/or MMRs could influence carcinogenesis and has been reported to be related to cancer pathophysiology [14,15]. Understanding how diet–microbiome metabolite and MMR interactions affect cancer biology could lead to dietary recommendations and interventions aimed at reducing cancer risk.

Recent advancements in high-throughput sequencing, metabolomics, and bioinformatics have propelled microbiome research forward, enabling more detailed and comprehensive analyses of microbial communities and their functional capacities [16]. Studies leveraging these technologies have begun to unravel the complex networks of host–microbiome interactions and their implications for cancer biology. For instance, metagenomic analyses have identified distinct microbial signatures associated with different types of cancer, while metabolomic profiling has revealed specific microbial metabolites that correlate with cancer risk and progression [17,18]. The current literature provides a wealth of information on the microbiome–microbiota–metabolite–cancer axis, but many questions remain unanswered [19]. Future research is needed to elucidate the precise mechanisms through which microbial metabolites influence cancer development, to identify the key microbial players and their metabolic products, and to explore the potential of manipulating the microbiome for cancer therapy. The pervasive influence of the microbiome across various organs underscores its significant potential in modulating cancer pathology. The microbiome’s dynamic interactions with host tissues suggest it plays a critical role not only in maintaining homeostasis but also in shaping disease processes, including cancer. Recent studies have highlighted how microbial communities contribute to the regulation of the tumor microenvironment across a diverse range of cancer types [20,21]. Furthermore, tumor cells are characterized by an abundance of receptors [22,23,24], including MMRs, that play critical roles in modulating various aspects of their behavior and interactions within the tumor microenvironment. These receptors, spanning a wide range of functional categories, allow tumor cells to sense and respond to external signals, often to their advantage in promoting growth, survival, and metastasis.

In this work, through an in silico, pan-cancer study of twenty-three cancer types, we delve into the current understanding of 107 MMR transcriptomic profiles and their roles in cancer biology. We examine the key microbial metabolites involved, their receptor-mediated mechanisms of action, and the implications of these interactions for cancer pathogenesis and therapy both on cancer cell lines and human tumor samples. We show how these receptors are co-expressed with known cancer hallmark genes (CHGs) and their influence on cancer hallmark pathways (CHPs), which involve the main mechanisms of cancer-cell survival. Our work, for the first time, addresses in bulk the role of MMRs in the hope that they will prove to be useful diagnostic, prognostic, and therapeutic biomarkers for several cancer types.

## 2. Materials and Methods

Our broad analyses employ a multipronged approach to unravel the role of MMRs across several types of cancer. Initially, through an extensive review of the existing literature, we identified 107 MMRs that interact with ten distinct categories of ligands, as detailed in Table 1. Following this identification, we conducted an in-depth investigation into the expression patterns of these MMRs within cancer cell lines and tumors derived from human patients. This dual strategy allowed us to correlate the presence and expression of MMRs with specific cancer types, providing valuable insights into their potential functions and implications in cancer progression and treatment.

### 2.1. Microbial Metabolite Receptors in Cancer Cell Lines

TPM (transcripts per million) expression data were obtained from the latest release of Broad Institute’s Cancer Cell Line Encyclopedia (CCLE 2019) (RRID:SCR_013836) [25] for 1019 cell lines. According to the CCLE consortium, RNA-seq reads were aligned using STAR 2.4.2a [26]. Gene-level read count values in TPM (transcripts per million) was quantified using RSEM v.1.2.22 [27]. These data were first quantile-normalized and subsequently aggregated per tissue-specific tumor in the R statistical language v.4.4.0 [28]. For each tissue type, the mean expression values (mean normalized TPM) of the corresponding MMRs were calculated. Additionally, these expression values of MMRs were grouped and analyzed by ligand type. In total, 26 tissue-specific cancers were analyzed: autonomic ganglia, biliary tract, bone, breast, central nervous system, cervix, endometrium, fibroblast, hematopoietic and lymphoid tissue, kidney, large intestine, liver, lung, esophagus, ovary, pancreas, pleura, prostate, salivary gland, skin, small intestine, soft tissue, stomach, thyroid, upper aerodigestive tract, and urinary tract. The formalized dataset was subset to include only the expression values for the MMRs under investigation, which were then subjected to summarizing analyses. A log2 transformation was applied to our summaries for visualization purposes only after the analyses were complete.

### 2.2. Microbial Metabolite Receptors in Human Cancer

As a source for our analyses, The Cancer Genome Atlas Program (TCGA) (RRID:SCR_003193) was selected to cover a variety of different cancers. To ensure that the data retrieved were comparable among our analyses and the results reproducible, we decided to use the uniformly processed RNAseq data provided via the recount3 R-package v.1.14.0 [29]. This allowed us to ensure data integrity and pre-processing.

Out of 33 cancer-specific projects included in TCGA, we selected 23 projects that include both solid-tumor and normal tissue samples to be able to perform differential gene expression (DGE) and correlation analyses, namely the datasets TCGA-THCA (Thyroid Carcinoma), TCGA-GBM (Glioblastoma Multiforme), TCGA-SARC (Sarcoma), TCGA-KIRC (Kidney Renal Clear Cell Carcinoma), TCGA-KIRP (Kidney Renal Papillary Cell Carcinoma), TCGA-KICH (Kidney Chromophobe), TCGA-PCPG (Pheochromocytoma and Paraganglioma), TCGA-PRAD (Prostate Adenocarcinoma), TCGA-CHOL (Cholangiocarcinoma), TCGA-LIHC (Liver Hepatocellular Carcinoma), TCGA-PAAD (Pancreatic Adenocarcinoma), TCGA-STAD (Stomach Adenocarcinoma), TCGA-COAD (Colon Adenocarcinoma), TCGA-READ (Rectum Adenocarcinoma), TCGA-HNSC (Head and Neck Squamous Cell Carcinoma), TCGA-ESCA (Esophageal Carcinoma), TCGA-THYM (Thymoma), TCGA-BRCA (Breast Invasive Carcinoma), TCGA-LUAD (Lung Adenocarcinoma), TCGA-LUSC (Lung Squamous Cell Carcinoma), TCGA-BLCA (Bladder Urothelial Carcinoma), TCGA-CESC (Cervical Squamous Cell Carcinoma and Endocervical Adenocarcinoma), and TCGA-UCEC (Uterine Corpus Endometrial Carcinoma). After filtering to keep only the solid-tumor samples and normal tissue controls, we were left with approximately 8500 tumor samples and 740 matched controls. All RNA sequencing read counts were processed using the transform_counts() function for each TCGA project to allow them to be used for downstream analysis.

### 2.3. MMR-Based Transcriptomic Profile of Cancer Types

To identify genes differentially perturbed in each cancer type, we used the DESeq2 v.1.44.0 R package (RRID:SCR_000154) [30]. The normal tissue samples were set as controls, while the solid tumor ones were used as the phenotype under investigation. All analyses were batch-corrected using the tcga.cgc_case_batch_number metadata provided by the recount3 package by integrating them in the DESeq2 formula. The results, including log2 fold changes and Benjamini–Hochberg false discovery rates (FDR), were saved into individual files for subsequent analyses and visualization in R.

To aggregate data and visualize over- and under- expression of MMRs in all cancer types, we employed the tidyverse v.2.0.0 [31], pheatmap v.1.0.12 (RRID:SCR_016418) [32], and ggplot2 v.3.5.1 (RRID:SCR_014601) [33] R packages. The data were grouped by cancer type and MMR HUGO symbol and summarized to create a heatmap highlighting the perturbed MMRs in each cancer type. Also, the mean MMR log2FoldChange values per ligand were calculated to create a heatmap of which ligand receptor types (according to Table 1) were over- (log2FoldChange > 1, FDR < 0.05) or under-expressed (log2FoldChange < −1, FDR < 0.05) in the 23 cancer types. Hierarchical clustering was performed using Euclidean distances. In addition, the same data were used to create Spearman correlations and calculate the asymptomatic *p*-values between each cancer type based on their MMR expression profiles using the Hmisc R package (doi: 10.32614/CRAN.package.Hmisc, Harrell Miscellaneous (RRID:SCR_022497)). 

### 2.4. Pan-Cancer Correlation of MMRs and Cancer Hallmarks

Along with the data previously obtained and analyzed, we acquired a list of 1245 unique genes involved in CHPs. The genes were obtained from three different sources: the Catalog of Somatic Mutations In Cancer (COSMIC) platform (RRID:SCR_002260) [34], the Integrative Onco Genomics platform (IntOGen) [35], and the work of Nagy et al. published in 2021 [36]. This was done to create a more modern list of hallmarks of cancer as established in the work of Hanahan and Weinberg [37]. We then extracted their log2FoldChange values from our original DGEA per cancer type without applying any restrictions, only filtering them for FDR < 0.05 to be able to capture any correlations, using Spearman’s correlation coefficient (Rho) calculations and their corresponding *p*-values between all MMRs and our list of CHGs. Finally, after mapping all the CHGs to the 10 categories of CHPs (evading growth suppressors, evading immune destruction, genome instability, replicative immortality, reprogramming energy metabolism, resisting cell death, sustained angiogenesis, sustaining proliferative signaling, tissue invasion and metastasis, and tumor promoting inflammation), we were able to correlate our MMRs with those processes. For each CHP, we calculated the median Spearman’s rho of all genes associated with that specific pathway and MMRs across all cancer types. This approach allowed us to capture the central tendency of correlations within each pathway. Finally, we kept the quantiles for the top and bottom 25% of all correlations. By doing so, we could systematically compare the behavior of genes in CHPs and the MMRs in the diverse spectrum of cancer types studied.

All relevant scripts used in these analyses and the production of the figures in this work can be found on GitHub (https://github.com/ndovro/MMR) (accessed on 11 December 2024).

## 3. Results

### 3.1. MMR Expression in Cancer Cell Lines

As described in our methodology, we used CCLE data to determine the mean expression of the MMRs under investigation in a variety of cancer cell lines grouped by tumor tissue. To fully understand these expression patterns, we examined three different questions: (1) Which MMR has the highest expression in each tissue? (2) For each MMR, what is the tissue it is most expressed in? (3) If we assume that high MMR expression translates to high ligand presence, then which tissue types can be associated with those ligands?

**Figure 1:**
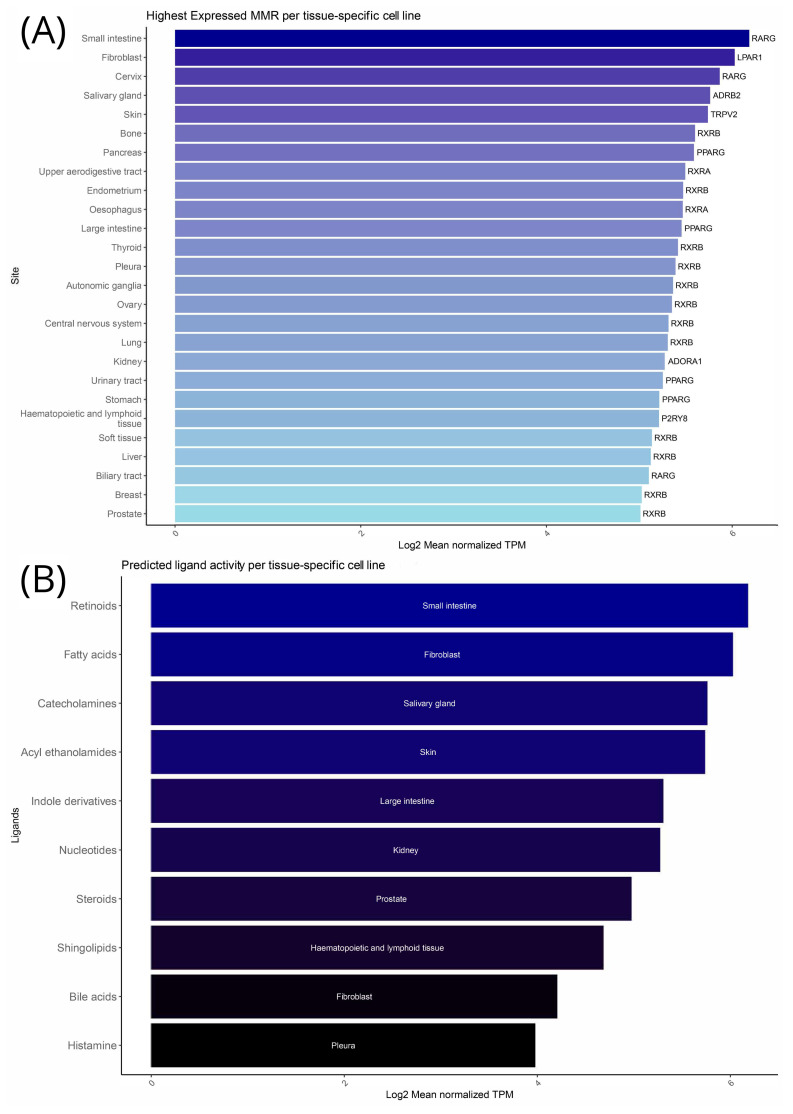
Microbial metabolite receptor (MMR) expression in the Cancer Cell Line Encyclopedia (CCLE) dataset. (**A**) MMR with the highest expression per tissue-specific cell line. (**B**) MMRs summarized by their ligand, showing which tissue-specific cell line they are highly expressed in.

Our results showed that in the small intestine, *RARG* (Retinoic Acid Receptor Gamma) show the highest expression, while fibroblasts from tumors predominantly express *LPAR1* (Lysophosphatidic Acid Receptor 1), highlighting a possible importance in tumor tissue formation. Again, in the cervical tumors, *RARG* is highly expressed, while the salivary gland tumor cells exhibit the highest expression of *ADRB2* (Adrenoceptor Beta 2), implicating its crucial function in glandular processes. The rest of the list expands to high expression of *TRPV2* (Transient Receptor Potential Vanilloid 2) in cancerous skin cells, *RXRB* (Retinoid X Receptor Beta) in bone, and *PPARG* (Peroxisome Proliferator-Activated Receptor Gamma) in pancreatic cancer. The tumor cells from the endometrium, esophagus, thyroid, and kidneys show dominant expressions of receptors like *RXRA* (Retinoid X Receptor Alpha)*, RXRB, PPARG,* and *ADORA1* (Adenosine A1 Receptor), underscoring their diverse physiological roles. It appears that the retinoid receptors’ (*RXRA, RXRAB,* and *RARG*) expression is highly involved in the majority of cell lines (Figure 1A). For a more detailed view, Supplementary Figure S1 illustrates the cancer cell line types in which each of our 107 MMRs is most prominently expressed.

When studying the per-ligand involvement, retinoids exhibited the highest mean receptor expression in the cancer cell lines of the small intestine, consistent with the high expression of RARG in the same cells. Fatty and bile acid receptors are predominantly expressed in fibroblasts, catecholamines show the highest involvement in the salivary gland cells, acyl-ethanolamides are mostly involved in the skin, and large intestine tumor cells exhibit high indole derivatives’ activity. Meanwhile, nucleotides show predominant expression patterns of their receptors in the kidney cells and steroids in the prostate, a fact that might correlate with the tissue’s role in steroid hormone processing. Finally, sphingolipids are active in hematopoietic and lymphoid tissues, whereas histamine is predominant in the pleura (Figure 1B). The observation that several tissues share common MMRs as the highest expressed genes, while ligands uniquely point to specific tissue types (except for bile and fatty acids), is remarkably interesting. It shows that common regulatory mechanisms may be at play across different cancer cell lines, yet ligand interactions provide a level of specificity that dictates tissue-specific functions. This dual pattern of shared MMR expression and unique ligand association underscores the complexity of gene regulation and suggests that while some genetic elements are universally important, ligand-specific signaling pathways fine-tune their roles to meet the distinct needs of individual tissues.

### 3.2. Pan-Cancer Differential MMR Expression

Understanding the transcriptomic alterations induced by cancer is crucial for elucidating the role of specific gene perturbations and their contributions to oncogenesis. By studying these changes, we hoped to gain insights into the complex molecular mechanisms underlying cancer development and progression. In our investigation, we focused on examining the differential expression of MMRs between tumor tissues and their corresponding normal adjacent tissues. This analysis aimed to identify potential diagnostic or therapeutic biomarkers among these receptors and their ligands.

The results revealed several MMRs with significant alterations in their expression levels. Some MMRs exhibited over-expression in tumor tissues, suggesting their potential role in promoting cancer cell survival and proliferation through enhanced microbial metabolite signaling, while others were found to be under-expressed, indicating a possible loss of function that may disrupt normal metabolic interactions and contribute to the oncogenic process. Some very interesting insights can be drawn from these results: *GPR84* (G protein-coupled receptor 84) is statistically significantly (log2FoldChange > 1, FDR < 0.05) upregulated in 14 out of the 23 cancer types, followed by *HTR1D* (5-hydroxytryptamine receptor 1D) in 11 and *HTR3A* (5-hydroxytryptamine receptor 3A), *GABRG2* (gamma-aminobutyric acid type A receptor subunit gamma2), *TRPM8* (transient receptor potential cation channel subfamily M member 8), *HTR2C* (5-hydroxytryptamine receptor 2C), and *P2RY6* (P2Y purinoceptor 6) in 10. Interestingly, *LPAR2* (lysophosphatidic acid receptor 2) is over-expressed in 17 cancer types but only manages to attain log2FoldChange > 1 in 8. On the other end of the spectrum, *ADRA1A* is statistically significantly downregulated in 17 cancer types, followed by *PPARGC1A* (peroxisome proliferator-activated receptor gamma coactivator 1-alpha) in 14; *RXRG* (retinoid X receptor gamma), *S1PR1* (sphingosine-1-phosphate receptor 1), and *CHRM2* (cholinergic receptor muscarinic 2) in 13; *AR* (androgen receptor) and *P2RX1* (purinergic receptor P2X 1) in 11; and *CNR1* (cannabinoid receptor 1), *ADRB1* (adrenoceptor beta 1), *HTR4* (5-hydroxytryptamine receptor 4), *NR1H4* (nuclear receptor subfamily 1 group H member 4), *ADRB3* (adrenoceptor beta 3), *ADRB2*, *ADRA1D* (adrenoceptor alpha 1D), and *P2RY14* (P2Y purinoceptor 14) in 10. *ADRA1A* in total appears to be the most engaged MMR during cancer, consistently downregulated. In addition, several MMRs appear to have a distinct behavior in different cancer types, like *CNR1*, which is statistically significantly downregulated in 10 cancer types and upregulated in five, while *HTR3A* does the opposite. Supplementary Table S1 shows for each MMR in how many of our studied cancer types it is upregulated or downregulated significantly (log2FoldChange > 1, log2FoldChange < −1, adjusted-*p* < 0.05).

**Figure 2:**
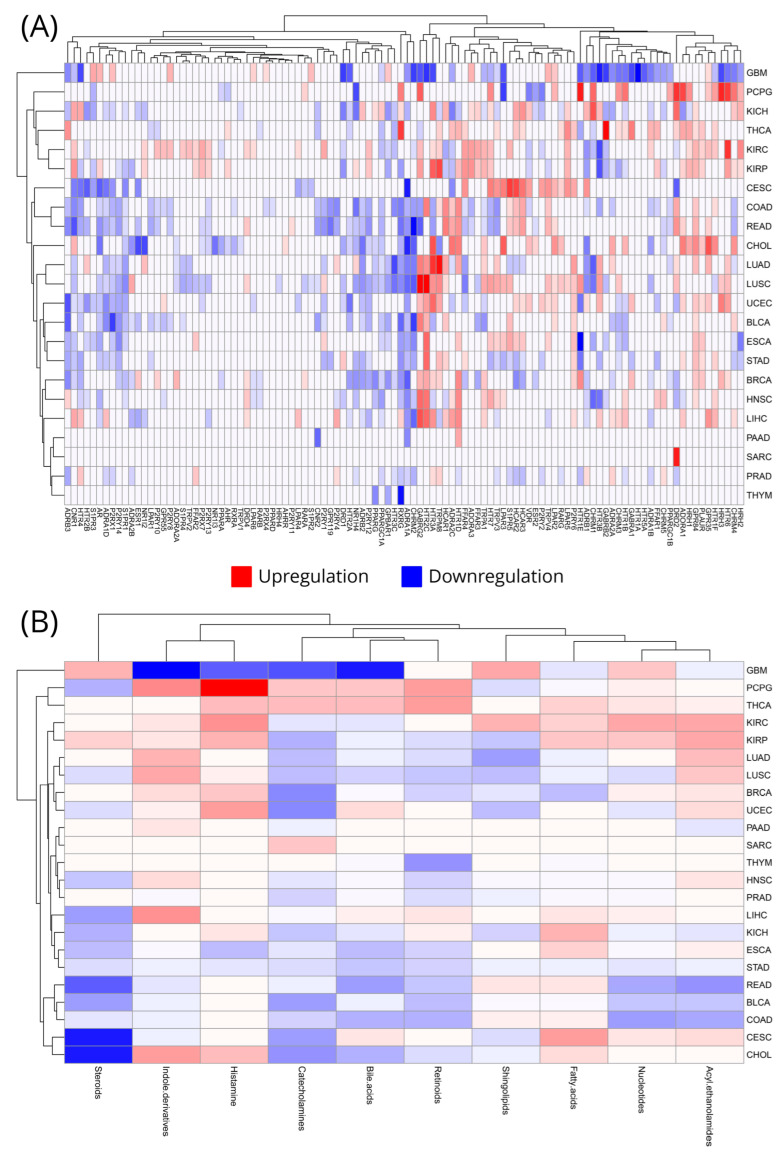
Differential expression of microbial metabolite receptors (MMRs) in a pan-cancer setting. (**A**) Each MMR’s dysregulation versus control samples per studied cancer type. (**B**) MMR expression dysregulation summarized by ligand type per studied cancer type. Red color signifies upregulation and blue downregulation.

Cancer specificity is another aspect we wanted to explore, and our results showed that *P2RY11* (Purinergic Receptor P2Y, G-Protein Coupled, 11) is only statistically significantly upregulated in rectal adenocarcinoma, while *NR1I3* (Nuclear Receptor Subfamily 1, Group I, Member 3) is uniquely downregulated in cholangiocarcinoma, *PPARD* (Peroxisome Proliferator-Activated Receptor Delta) in colorectal adenocarcinoma, and *HTR1A* (5-Hydroxytryptamine Receptor 1A) in glioblastoma. Interestingly, *RXRB,* previously highlighted in cancer cell lines, did not present with a statistically significant fold regulation in any cancer type. A graphical representation of the totality of our results, including those described here, can be found in Figure 2A. We also provide hierarchical clustering of the MMRs according to their expression patterns across all cancer types and vice versa.

When trying to decipher the role of any kind of receptor it is always useful to put them in perspective with their ligands. The MMRs are no exception, so we decided to also aggregate them according to their ligands and for each group, as described in the methodology, to see how they participate in the different cancer types. Catecholamines, bile acids, retinoids, and steroids receptors present with an increased tendency to be downregulated in most cancer types, with the latter exhibiting the highest effect, especially in cholangiocarcinoma and cervical squamous cell cancer. Colorectal and breast cancer subtypes appear to contribute to this trend, presenting mainly with downregulated MMR groups. However, there are exceptions to this, such as in thyroid carcinoma, where these MMR groups show upregulation. In contrast, receptors of indole derivatives, histamines, and fatty acids mainly present with an upregulation in most cancer types, perhaps with the exception of glioblastoma. It appears that thyroid carcinoma, exclusively, in this case, and the two renal carcinoma subtypes (clear cell and papillary cell) are particularly sensitive to the upregulation of several MMR groups. The complete effect of these groupings on different cancer types can be seen in Figure 2B along with hierarchical clustering of the cancer types according to their ligand grouping expression.

In the search for novel biomarkers, in addition to tendencies, the magnitude of changes also plays an important role. The analysis of the cancer dataset reveals distinct patterns of upregulation and downregulation of MMRs across various cancer types. In TCGA-BLCA, notable upregulations include *GABRG2* with a fold change of 15.9, *HTR2C* at 5.2, and *S1PR5* (Sphingosine-1-Phosphate Receptor 5) at 4.2, while significant downregulations are seen in *CHRM2* (−49.8-fold), *P2RX1* (−47.3-fold), and *ADRB3* (−28.7-fold). For TCGA-BRCA, *HTR1E* (5-Hydroxytryptamine Receptor 1E) is upregulated by 12.6-fold and *HTR1D* by 11.4-fold, with downregulations observed in *ADRA1A* (−26.1-fold) and *HCAR2* (Hydroxycarboxylic Acid Receptor 2) (−10.8-fold). In TCGA-CESC, *S1PR5* is highly upregulated at 49.9-fold and *HCAR2* at 37.5-fold, contrasted with significant downregulations in *ADRA1A* (−96.2-fold) and *HTR2B* (5-Hydroxytryptamine Receptor 2B) (−28.4-fold). TCGA-CHOL (Cholangiocarcinoma) shows upregulations of *HTR3A* (35.1-fold) and *GPR35* (G Protein-Coupled Receptor 35) (33.9-fold), with downregulations in *ADRA1A* (−74.3-fold) and *ESR1* (Estrogen Receptor 1) (−43.2-fold). For TCGA-COAD, *HTR2C* is upregulated by 15.1-fold and *HCAR1* (Hydroxycarboxylic Acid Receptor 1) by 11.0-fold, while downregulated receptors include *NR1H4* (−17.3-fold) and *RXRG* (−15.7-fold). In TCGA-ESCA, *HTR2C* is upregulated by 22.7-fold and *S1PR5* by 6.2-fold, with notable downregulations in *HTR1E* (−203.1-fold) and *HRH2* (Histamine Receptor H2) (−14.2-fold). TCGA-GBM exhibits upregulations in *AR* and *S1PR3* (Sphingosine-1-Phosphate Receptor 3), both at 5.5-fold, and downregulations in *HTR1A* (−186.3-fold) and *HTR3B* (5-Hydroxytryptamine Receptor 3B) (−95.4-fold). TCGA-HNSC shows upregulations in *GABRG2* (23.7-fold) and *HTR2C* (19.6-fold), while *CHRM1* (Cholinergic Receptor Muscarinic 1) and *HTR3B* are downregulated by −20.4- and −18.0-fold, respectively. In TCGA-KICH, *CHRM1* is upregulated by 32.0-fold and *DRD2* (Dopamine Receptor D2) by 22.9-fold, while *NR1H4* and *DRD1* (Dopamine Receptor D1) are downregulated by −16.2- and −13.9-fold, respectively. TCGA-KIRC shows *HTR6* (5-Hydroxytryptamine Receptor 6) upregulated by 56.9-fold and *HRH2* by 11.4-fold, with downregulations in *HTR3B* (−38.9-fold) and *ADRB1* (Adrenoceptor Beta 1) (−9.1-fold). For TCGA-KIRP, *TRPM8* is upregulated by 38.6-fold and *HTR3A* by 27.4-fold, with downregulations in *HTR3B* (−36.8-fold) and *DRD1* (−9.8-fold). In TCGA-LIHC, *GABRG2* is upregulated by 35.6-fold and *HTR2C* by 20.1-fold, while *CHRM2* and *ADRA1A* are downregulated by −22.5- and −8.8-fold, respectively. TCGA-LUAD exhibits a significant upregulation in *TRPM8* (107.7-fold) and *HTR3A* (63.4-fold), with downregulations in *HTR3C* (5-Hydroxytryptamine Receptor 3C) (−43.4-fold) and *CHRM1* (−28.4-fold). TCGA-LUSC shows *HTR2C* highly upregulated at 203.8-fold and *GABRG2* at 150.4-fold, with notable downregulations in *CHRM2* (−29.6-fold) and *ADRA1A* (−21.4-fold). For TCGA-PAAD, *HTR1D* is upregulated by 6.1-fold, while *CNR2* (Cannabinoid Receptor 2) is downregulated by −23.2-fold. TCGA-PCPG exhibits upregulations in *HTR1E* (91.6-fold) and *HRH3* (Histamine Receptor H3) (70.5-fold), with significant downregulations in *LPAR3* (Lysophosphatidic Acid Receptor 3) (−55.0-fold) and *NR1H4* (−37.7-fold). In TCGA-PRAD, *FFAR2* (Free Fatty Acid Receptor 2) is upregulated by 3.5-fold and *HTR4* by 3.1-fold, with downregulations in *ADRB3* (−5.8-fold) and *CHRM2* (−4.5-fold). TCGA-READ shows upregulations in *HCAR1* (15.8-fold) and *HTR1D* (9.9-fold), with *CHRM2* (−134.0-fold) and *CNR1* (−35.4-fold) notably downregulated. TCGA-SARC only exhibits a significant upregulation in *DRD2* by 102.7-fold. TCGA-STAD shows *HTR2C* upregulated by 24.1-fold and *TRPM8* by 4.4-fold, with downregulations in *HTR1E* (−16.4-fold) and *RXRG* (−9.9-fold). For TCGA-THCA, *GABRB2* (Gamma-Aminobutyric Acid Type A Receptor Subunit Beta2) is upregulated by 159.5-fold and *RXRG* by 47.2-fold, while *HTR1E* and *HTR3C* are downregulated by −6.3- and −4.5-fold, respectively. In TCGA-THYM, we did not observe any statistically significant upregulations, but *RXRG* is downregulated by −120.3-fold and *PPARG* by −11.6-fold. Finally, in TCGA-UCEC, *HTR3A* is upregulated by 24.2-fold and *HRH3* by 15.6-fold, with notable downregulations in *ADRB3* (−34.9-fold) and *HTR1E* (−19.1-fold).

**Figure 3:**
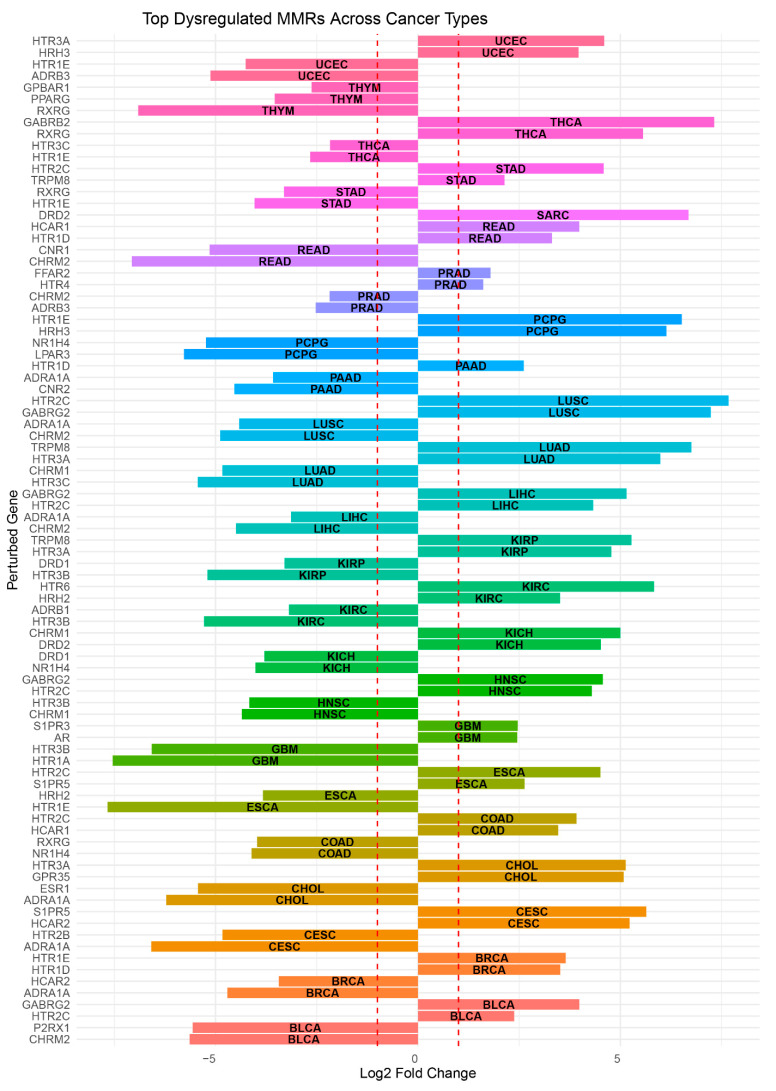
Top dysregulated microbial metabolite receptors (MMRs) per studied cancer type. X-axis represents log2FoldChange values, while the dashed red lines signify log_2_FoldChange of ±1.

These results highlight some very significant fold changes in specific MMRs, providing fertile ground for future works, and they are summarized in Figure 3, where we present the top two upregulated and downregulated MMRs per cancer type, with the exception of TCGA-THYM, in which we found no upregulation of MMRs, and TCGA-SARC along with TCGA-PAAD, in which we found only one upregulated MMR. We also provide Table 2, which presents the results described here in a more accessible form.

### 3.3. Correlating Cancer Types Based on Their MMR Expression

**Figure 4:**
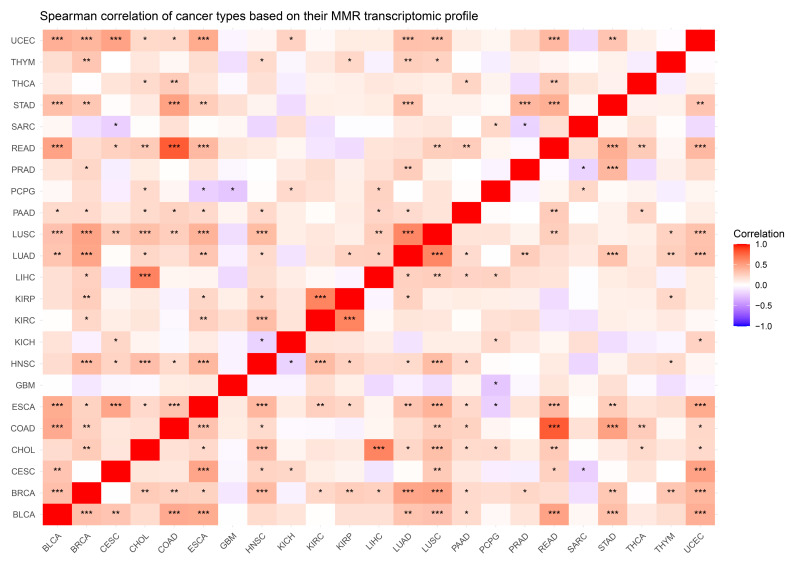
Spearman Correlation Heatmap showcasing the relationship between cancer types based on MMR expression profiles. Red indicates a positive correlation, while blue highlights inverse correlations. (* *p*-value < 0.05; ** *p*-value < 0.01; *** *p*-value < 0.001).

An important aspect of biomarker discovery is the ability to identify specific conditions and their subtypes either uniquely or to find overlaps between them suitable for therapeutic intervention. In that spirit, we wanted to explore how similar or dissimilar the cancer types under investigation are when they are characterized only by their MMR transcriptomic profiles and stripped of any other contributing factors. In Figure 4, we depict those correlations between cancer types and observe medium to strong positive and negative relationships. In most cases, the correlation is positive, without showing strong tendencies (−0.5 < Spearman’s rho < 0.5); however, there are cancer types that correlate strongly (Spearman’s rho > 0.7, *p*-value < 0.001). The pairs of Kidney Renal Clear Cell Carcinoma–Kidney Renal Papillary Cell Carcinoma as well as Lung Squamous Cell Carcinoma–Lung Adenocarcinoma and Bladder Urothelial Carcinoma–Uterine Corpus Endometrial Carcinoma appear highly correlated, perhaps due to their common tissue origin, further reinforcing the tissue-specific MMR transcriptomic expression profile hypothesis. However, Kidney Chromophobe does not share that strong relationship with its same-origin cancer types. Interestingly, the pair of Liver Hepatocellular Carcinoma–Cholangiocarcinoma is highlighted in the results: two cancer types of tissues in close proximity and interaction in the body. Unsurprisingly, Colon Adenocarcinoma and Rectum Adenocarcinoma also present with similar MMR profiles, strengthening the customary practice in the literature to be studied as one cancer type (colorectal cancer). Supplementary Table S2 contains all *p*-values for the presented pairwise correlations.

### 3.4. Cancer Hallmarks and MMRs

Identifying the interactions between MMRs and CHGs is important for starting to understand how the perturbation caused by microbial metabolites in the tumor microenvironment affects or combats tumor initiation and progression.

**Figure 5:**
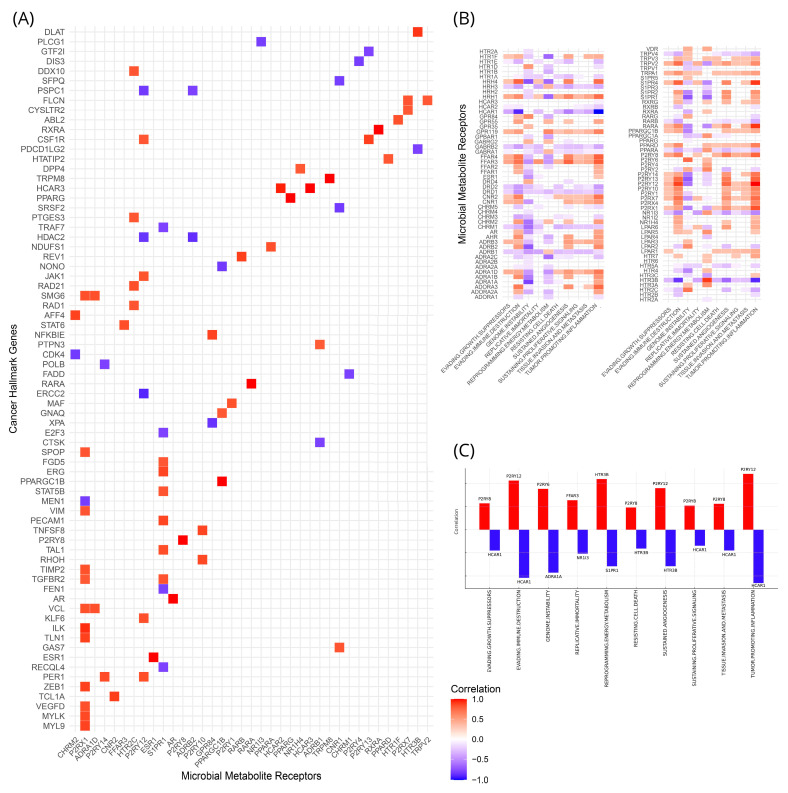
(**A**) Pairwise Spearman correlation of cancer hallmark genes (CHGs) and microbial metabolite receptors (MMRs) based on their expression. All correlations exhibit Spearman’s rho lower than −0.8 or higher than 0.8 and *p*-values lower than 10^−10^. (**B**) Pairwise Spearman correlation of MMRs when the CHGs are aggregated into cancer hallmark pathways (CHPs) (**C**) Summary of the strongest correlations between MMRs and CHPs.

As stated in our methodology, we constructed a combined list of 1245 genes associated with specific tumor-promoting and survival mechanisms (Supplementary Table S3). We assessed the correlations of both the expression of these genes and their aggregation in ten specific CHPs with the expression of our MMRs across all cancer types. This resulted in over 129.000 pairwise combinations between CHGs and MMRs (Supplementary Table S4). When filtered to only keep those pairs with a Spearman’s rho greater than 0.8 and lower than −0.8, we obtained a more manageable dataset of 81 pairwise comparisons (59 positive and 22 negative) with correlation *p*-values < 10^−10^. A total of 32 unique MMRs and 72 unique CHGs contributed to these pairs. The total interactions are shown in Figure 5A. The only pairs that achieved a rho of 1.00 were nine MMRs that intersected with the CHGs, namely *ESR1*, *AR*, *P2RY8* (Purinergic Receptor P2Y, G-Protein Coupled, 8), *PPARGC1B* (Peroxisome Proliferator-Activated Receptor Gamma Coactivator 1 Beta), *RARA* (Retinoic Acid Receptor Alpha), *PPARG*, *HCAR3* (Hydroxycarboxylic Acid Receptor 3), *TRPM8*, and *RXRA*. The five highest positive correlations were shown for the *HCAR2*–*HCAR3* (0.96), *P2RX1*–*ILK* (Integrin Linked Kinase) (0.93), *HTR3B*–*DLAT* (Dihydrolipoamide S-Acetyltransferase) (0.91), *CNR2*–*TCL1A* (T-Cell Leukemia/Lymphoma 1A) (0.90), and *P2RY13* (Purinergic Receptor P2Y, G-Protein Coupled, 13)–*CSF1R* (Colony Stimulating Factor 1 Receptor) pairs of MMR-CHG. On the other end, showing the strongest negative correlations were the pairs of *P2RY12* (Purinergic Receptor P2Y, G-Protein Coupled, 12)–*ERCC2* (Excision Repair Cross-Complementing Rodent Repair Deficiency, Complementation Group 2) (−0.91), *ADRB2*–*HDAC2* (Histone Deacetylase 2) (−0.88), *GPR84*–*XPA* (Xeroderma Pigmentosum, Complementation Group A) (−0.87), *P2RY12*–*HDAC2* (Histone Deacetylase 2) (−0.86), and *PPARGC1B*–*NONO* (Non-POU Domain Containing, Octamer-Binding) (−0.84). The most active MMRs were *P2RX1*, with 12 positive and one negative correlation, followed by *S1PR1*, with six positive and four negative correlations.

When we aggregated the CHGs into CHPs, we observed a plethora of interactions between our MMRs and specific pathways (Figure 5B). The highest positively and negatively correlated MMRs per hallmark pathway can be seen in Figure 5C. In more detail, the top three highest correlated MMRs in descending order for evading growth suppressors are *P2RY8*, *FFAR3*, and *HRH1*; for evading immune destruction *P2RY8, FFAR3,* and *HRH1*; for genome instability *P2RY6*, *HTR2C*, and *LPAR2*; for replicative immortality *FFAR3*, *TRPV2*, and *S1PR4* (Sphingosine-1-Phosphate Receptor 4); for reprogramming energy metabolism *HTR3B*, *P2RY2* (Purinergic Receptor P2Y2), and *GPR119* (G Protein-Coupled Receptor 119); for resisting cell death *P2RY8, HRH1,* and *TRPV2*; for sustained angiogenesis *P2RY12*, *FFAR3*, and *P2RY8*; for sustaining proliferative signaling *P2RY8, FFAR3,* and *HRH1*; for tissue invasion and metastasis *P2RY8, ADRA1D*, and *HRH1*; and for tumor promoting inflammation *P2RY12, RARA*, and *S1PR4*. As for the negative correlations, again in descending order, we showed *NR1I3*, *HTR3B,* and *HCAR1* for evading growth suppressors; *NR1I3, HTR3B,* and *HCAR1* for evading immune destruction; *P2RY13*, *S1PR1*, and *ADRA1A* for genome instability; *ADRB1*, *HTR3B*, and *NR1I3* for replicative immortality; *HRH1, HTR1F* (5-Hydroxytryptamine Receptor 1F), and *S1PR1* for reprogramming energy metabolism; *ADRB1, CHRM1*, and *HTR3B* for resisting cell death; *NR1I3, HCAR1,* and *HTR3B* for sustained angiogenesis; *GABRB2*, *ADRB1*, and *HCAR1* for sustaining proliferative signaling; *ADRB1, NR1I3,* and *HCAR1* for tissue invasion and metastasis; and *NR1I3, HTR3B,* and *HCAR1* for tumor promoting inflammation. It is apparent from these results that nucleotide receptors dominate the positive correlations, while fatty acids receptors have a reversing role.

## 4. Discussion

To identify and assess the role of MMRs in cancer and provide new potential biomarkers of diagnosis and therapy, this study establishes clear transcriptomic relationships between these MMRs and cancer hallmarks, offering a unique perspective on the mechanisms in which the former contribute to cancer biology. Furthermore, we clearly highlight significant MMR imbalance in a pan-cancer context, revealing altered expression of multiple MMR genes common across various cancer types, as well as distinct associations between MMRs and specific cancer tissues. Although the role of microbiota-derived metabolic products in carcinogenesis has been increasingly gaining focus during the last decade [38], there is a paucity in the literature studying the potential interplay of these molecules with host receptors, while the studies that do exist tend to focus on specific malignancies [39,40].

To elucidate the landscape of MMR expression across cancer types and establish if they are expressed in specific cells, we first focused our attention on well-characterized cell lines that represent each tumor tissue and observed that in most cases the retinoid receptor genes, namely *RARG*, *RXRA*, and *RXRB*, are notably expressed. This finding seems rather contradictory considering that retinoid-induced signaling through RXR receptors hinders the growth of cancer cells, and retinoid agonists have been tested and used as cancer treatments [41]. However, recent evidence indicates that paracrine retinoid signaling in the tumor microenvironment exerts pro-tumorigenic effects through immune suppression [42], which could explain the high expression tolerance of retinoid receptors in cancer cells, as it ultimately proves beneficial to their survival. This analysis also provided us with a map of tissues in which specific receptor types appear to be expressed, highlighting their variety. For example, we identified the small intestine as the tissue with the highest expression of receptors for retinoids. In fact, retinoid signaling is essential for small intestine epithelial cell proliferation and differentiation, a physiological process with tumor-suppressive functions [43]. However, as both classes of retinoid receptors, RARs and RXRs, are known to act as homodimers or heterodimers with other nuclear receptors, such as PPARs and VDR, it could be possible that retinoid signaling in these cells could have a dual role, promoting carcinogenesis under the influence of different transcription partners [44].

Expanding our investigation on tumor tissue samples, we were able to highlight and investigate several pan-cancer observations for the selected MMRs. By studying their transcriptomic differences during cancer, we highlighted many potential diagnostic and/or therapeutic intervention targets. Among those we identified as globally dysregulated in cancer, the medium-chain fatty acid receptor *GPR84* was significantly upregulated in fourteen out of the twenty-three cancer types examined, highlighting this receptor as a promising biomarker. This observation can be supported by recent studies that have demonstrated a tumorigenic effect of *GPR84* in both esophageal cancer via attenuation of the antitumor CD8^+^ T-cell response [45] as well as in prostate cancer via its activation by the metabolite caprylic acid [46]. In addition, its expression in macrophages has been linked to enhanced phagocytic activity of cancer cells [47,48]. These results indicate a mixed role of *GPR84* in cancer, probably reflecting the various cell-dependent responses to its activation, leading to a fine balance between tumor growth and destruction.

Moreover, the three serotonin receptors *5-HTR1D*, 2C, and 3A showed significant upregulation in most cancer types we studied, in accordance with the literature in which they are reported upregulated in a few types of malignancies. The *5-HTR1D* receptor has been gaining attention as an important oncogene in gastrointestinal tumors, and its inhibition has been reported to exert antiproliferative effects in gastric [49], pancreatic [50], and colorectal cancer [51], rendering it a potential therapeutic target for these malignancies. Furthermore, both *5-HTR1D* and 2C were shown by Zhan et al. to be upregulated in breast cancer patients compared to healthy controls in different datasets [52], and *5-HTR2C* may also play a role in epithelial-to-mesenchymal transition and cancer metastasis [53]. Finally, the *5-HTR3A* receptor has also been reported to facilitate the development and progression of both colorectal and non-small-cell lung cancer through NLRP3 and Wnt3A/β-catenin signaling, respectively [54,55], thus confirming its role in carcinogenesis. In addition, we found that the serotonin receptors were also amongst the genes with the most dysregulated expression in cancer tissues versus controls, as the *5-HTR1E* receptor demonstrated −203-fold regulation in Esophageal Carcinoma and +91-fold in Pheochromocytoma and Paraganglioma, the *5-HTR1A* −186-fold in Glioblastoma Multiforme, and the *5-HTR2C* +203-fold in Lung Squamous Cell Carcinoma. These results indicate high involvement of the serotonin receptors in neoplasia in general, rendering promising disease biomarkers as well as candidates for therapeutic development.

On the other hand, we showed that the *ADRA1A* receptor gene was the most frequently downregulated gene in cancer (as shown in Supplementary Table S1), while it exhibited its greatest downregulation of −96-fold in Cervical Squamous Cell Carcinoma and Endocervical Adenocarcinoma. This aligns with recent studies that demonstrate that α1-adrenoreceptor under-expression in hepatocellular carcinoma, both through micro-RNA silencing [56] and promoter hypermethylation [57], influences tumor development and that *ADRA1A* downregulation in the prostate highly correlates with a prostate cancer diagnosis [58]. Similar to other findings, the expression of α1-adrenoreceptor in steroid-independent prostate cancer conferred protection to apoptosis and was associated with malignant transformation in pathology samples [59], thus suggesting a divergent role of this receptor depending on tumor phenotype. Our results, combined with previous knowledge, pinpoint *ADRA1A* as a potential tumor suppressor gene in various tissues and indicate a possible role in cervical cancer development that has not yet been described.

Furthermore, several species of gut microflora have been documented to influence cancer progression through metabolite–receptor interactions, providing direct evidence for a microbiome–cancer axis. These interactions have been demonstrated to drive cancer progression and metastasis through mechanisms including the activation of AhR signaling by products of tryptophan metabolism from *Lactobacillus* species [60] and the production of formate from *Fusobacterium nucleatum* [61]. Additionally, they can influence the response to immunotherapy through the activation of the ADRA2A receptor by inosine [62].

Apart from their expression in cancer cells, several MMR genes are also expressed in immune cells in the tumor microenvironment. For example, a GPR84^+^ subset of myeloid-derived suppressor cells in the tumor microenvironment of esophageal cancer can promote CD8^+^ T-cell senescence via the transfer of this receptor and activation of the p53 pathway [63]. Furthermore, activation of HCAR1 by breast cancer cell-secreted lactate in tumor-associated dendritic cells has been shown to disrupt antigen presentation, thus aiding in immune evasion of cancer cells [64]. The expression of MMRs in the immune cell component of the tumor microenvironment is an area of ongoing research, and by utilizing sequencing data from whole tissue, the differentially expressed MMRs that we have highlighted in this study could also pose promising targets for immune regulation in the tumor microenvironment.

Cancer hallmark genes and pathways have been a staple in cancer research trying to explain carcinogenesis, metastasis, and tumor growth. Correlating the expression of MMRs with these molecules and their functions allows us to put into perspective the significance of the former and allows future investigations of therapeutically disturbing these relationships. For example, we showed that the P2Y family of receptors exhibits the strongest positive correlation with most cancer hallmark pathways. Specifically, *P2RY8*, a recently de-orphanized receptor responding to the extracellular molecular S-geranylgeranyl-L-glutathione (GGG) [65], exhibited a high positive correlation six cancer hallmark pathways related to cell survival, proliferation, immune evasion, and metastasis. The *P2RY8* gene is known to be highly mutated in both hematologic malignancies, diffuse large B-cell lymphoma, and Burkitt lymphoma, driving their pathogenesis [66], and recent evidence also implicated its expression in the development of colorectal carcinoma through promoting the proliferation and migration of cancer cells [67]. Our findings regarding its over-expression in solid tumors indicate that it could also play a role in the development of breast cancer and glioblastoma, probably through similar mechanisms, rendering this receptor a putative therapeutic candidate.

Furthermore, we also showed several positive correlations of another purinergic receptor, the *P2RX1*, with several CHGs, the strongest of which being with the *ILK*. This kinase-coding gene is known to promote the development of gastric carcinoma [68,69] as well as the proliferation and platin-resistance phenotype of ovarian cancer [70] through modulation of various signaling pathways, including the Hippo-YAP, Wnt, and NF-κβ. Even though the *P2RX1* gene has been identified as a prognostic factor for various malignancies with ambiguous results [71,72,73], to our knowledge, there has been no previous association between these genes and *ILK,* pinpointing to a novel axis for therapeutic targeting. The MMR with the second greatest association with cancer hallmark genes was *S1PR*, coding for a sphingosine-1-phasphate receptor most known for its immune modulating properties, which are currently being used into clinical practice against ulcerative colitis and multiple sclerosis [74]. In support of our data that indicate a high involvement of this gene in carcinogenesis, this receptor was recently implicated in the development of both hematologic malignancies [75] and solid tumors such as ovarian [76] and esophageal cancer [77], strengthening the notion that it could present a potent oncogene and raising some concerns about possible long-term side effects of its therapeutic modulation.

The hydroxycarboxylic acid receptor *HCAR1* was also one of the most frequently and strongly correlated MMRs, showing a negative correlation with six CHPs. This association could be the result of metabolic reprogramming in cancer cells, as hypoxia and anaerobic glycolysis have been shown to promote their stemness and migration [78]. Indeed, *HCAR1* is a known modulator of cell metabolism, and its activation leads to a switch towards oxidative phosphorylation in cancer cells, contributing to the induction of a “quiescence” phenotype, together with the effects of its ligand, lactate [79]. Moreover, there has been evidence that *HCAR1* may also play a role in restricting the growth of tamoxifen-resistant breast cancer cells by altering fatty acid metabolism [80]. However, diverting from our observations, *HCAR1* activation has been reported to augment the proliferation, survival, and metastasis of various cancer cell lines, including colorectal [81], breast [82], ovarian [83], pancreatic [84], hepatocellular [85], and glioblastoma [79], thus marking this MMR as an oncogene. Conclusively, the strong negative correlation of this MMR with several cancer hallmark pathways observed in our data could be the sum of several divergent functions of this receptor, a finding warranting further investigation.

This study is based entirely on in silico analyses, without in vitro or ex vivo validation. The primary limitation stems from the substantial number of tumor samples required for experimental validation, given the broad spectrum of cancers and MMRs investigated. However, by leveraging computational approaches, we have laid a robust foundation for future, more focused experimental research. We have tried to alleviate this by using preprocessed data from well-known and tested sources to ensure the robustness of our analyses and findings. In addition, throughout this work, to translate in silico MMR expression in cancer tissue into metabolite abundance, we had to make the hypothesis that upregulated MMR expression correlates with an increased ligand abundance and vice versa. This limitation, however, is supported by evidence that MMR expression belonging to both the GPCR and the nuclear receptor classes can be modulated by their ligand metabolites, resulting in increased expression [79,86], However, the opposite is also true, as prolonged activation of GPCRs is known to lead to receptor internalization through arresting recruitment [87], and so, whether ligand accumulation leads to up- or downregulated expression depends on specific MMR properties and cell environment. Nevertheless, by following this simplification, we were able to make interesting observations regarding ligand class abundance and their probable role in carcinogenesis. One striking example is the strong positive correlation that we detected between histamine and several malignancies, including kidney, thyroid carcinomas, and, especially, the neuroendocrine tumors pheochromocytoma and paraganglioma. Currently, a growing body of literature is exploring the therapeutic potential of histaminergic signaling in cancer, with promising reports of cytostatic effects of histamine inhibition on colorectal cancer cells [88] and a putative role in aiding immunotherapy in hepatocellular carcinoma [89]. Together with the wide variety of histamine-producing bacteria in the gut microbiota [90], we suggest a strong role of histamine as a potential therapeutic target for a variety of neoplasms, such as pheochromocytoma, introducing it as a novel microbiota–metabolite-based therapy.

Further wet lab experiments building on these findings could play a crucial role in validating the in silico data and paving the way for designing targeted studies to explore the role of microbial metabolite receptors in cancer biology—a highly complex yet promising field of research. The first step would involve confirming and characterizing the expression of microbial metabolite receptors in tumor tissues. This includes identifying the specific microbial metabolites that interact with these receptors on cancer cells. By employing cancer cell lines and conducting in vitro experiments, researchers could delve deeper into how these metabolites influence cancer cell biology. These studies would focus on elucidating the signaling pathways that are activated or inhibited following the engagement of microbial metabolite receptors, providing insights into the molecular mechanisms underlying these interactions. Building upon this foundational understanding, subsequent research could utilize both in vitro and ex vivo models to investigate the potential for therapeutic interventions. For instance, identifying strategies to inhibit carcinogenesis-related actions mediated by microbial metabolite receptors might uncover novel therapeutic targets. These investigations could open new avenues for developing anti-cancer therapies, particularly those that modulate the tumor microenvironment or disrupt cancer-promoting signals initiated by microbial metabolites. Through this integrated approach, researchers can bridge computational predictions with experimental validation, advancing the field toward translational applications.

## 5. Conclusions

The aberrant expression of MMRs is widespread across solid malignancies, with each tissue type exhibiting unique profiles of ligand specificity. Our analysis of solid tumors, based on their MMR-related transcriptomic profiles, highlights the intricate nature of cancer biology and the distinct roles these receptors play in tissue-specific signaling pathways involved in cancer development. The gut microbiota, through the production of diverse metabolites, implicating gut–organ axes, plays a crucial role in either promoting or inhibiting the activation of these receptors, thereby significantly influencing cancer progression and tumor microenvironment dynamics. The strong correlation between specific MMRs, such as HTRs, P2Y, and HCARs, and hallmark cancer pathways emphasizes the existence of a microbiota–cancer axis. This axis represents a potential target for novel therapeutic strategies, as modulating the interaction between the microbiota and these receptors could lead to innovative treatments or even preventative measures against cancer. By leveraging the gut microbiota’s ability to influence MMR activity, researchers may unlock new avenues for cancer intervention that are both effective and specific to the patient’s tumor profile.

## Figures and Tables

**Table 1:** List of microbial metabolite receptors categorized by their ligands.

Fatty Acids	Indole Derivatives	Nucleotides	Bile Acids	Catecholamines	Acyl-Ethanolamides	Retinoids	Sphingolipids	Histamine	Steroids
FFAR1	AHR	ADORA1	GPBAR1	DRD1	TRPV1	RARA	S1PR1	HRH1	AR
FFAR2	AHRR	ADORA2A	CHRM1	DRD2	TRPV2	RARB	S1PR2	HRH2	ESR1
FFAR3	GPR35	ADORA3	CHRM2	DRD3	TRPV3	RARG	S1PR3	HRH3	ESR2
FFAR4	HTR1A	P2RY1	CHRM3	DRD4	TRPV4	RXRA	S1PR4	HRH4	
HCAR1	HTR1B	P2RY2	CHRM4	ADRA1A	TRPA1	RXRB	S1PR5		
HCAR2	HTR1D	P2RY4	CHRM5	ADRA1B	TRPM8	RXRG			
HCAR3	HTR1E	P2RY6	NR1H4	ADRA1D	CNR1				
GPR84	HTR1F	P2RY8	VDR	ADRA2A	CNR2				
PPARA	HTR2A	P2RY10	NR1I2	ADRA2B	GPR55				
PPARD	HTR2B	P2RY11	NR1I3	ADRA2C	GPR119				
PPARG	HTR2C	P2RY12	GABRA1	ADRB1					
PPARGC1A	HTR3A	P2RY13	GABRB2	ADRB2					
PPARGC1B	HTR3B	P2RY14	GABRG2	ADRB3					
LPAR1	HTR3C	P2RX1							
LPAR2	HTR4	P2RX4							
LPAR3	HTR5A	P2RX7							
LPAR4	HTR6								
LPAR5	HTR7								
LPAR6									

**Table 2:** Top dysregulated MMRs per cancer.

Cancer Dataset	Upregulated MMRs (Fold Regulation)	Downregulated MMRs (Fold Regulation)
TCGA-BLCA (Bladder Urothelial Carcinoma)	GABRG2 (15.9), HTR2C (5.2), S1PR5 (4.2), HCAR1 (3.2), P2RY6 (2.4)	CHRM2 (−49.8), P2RX1 (−47.3), ADRB3 (−28.7), RXRG (−23.1), ADRA1D (−12.3)
TCGA-BRCA (Breast Invasive Carcinoma)	HTR1E (12.6), HTR1D (11.4), HTR2C (6.5), ADORA2A (4.9), HRH3 (4.9)	ADRA1A (−26.1), HCAR2 (−10.8), PPARG (−10.6), ADRB3 (−10.4), RXRG (−9.9)
TCGA-CESC (Cervical Squamous Cell Carcinoma and Endocervical Adenocarcinoma)	S1PR5 (49.9), HCAR2 (37.5), HCAR3 (19.4), HTR7 (13.1), LPAR3 (13.0)	ADRA1A (−96.2), HTR2B (−28.4), DRD2 (−27.4), AR (−24.8), HTR4 (−19.5)
TCGA-CHOL (Cholangiocarcinoma)	HTR3A (35.1), GPR35 (33.9), LPAR3 (26.5), ADRA2C (16.5), HTR1D (14.9)	ADRA1A (−74.3), ESR1 (−43.2), NR1I2 (−41.8), CHRM2 (−28.8), AR (−18.4)
TCGA-COAD (Colon Adenocarcinoma)	HTR2C (15.1), HCAR1 (11.0), HTR1D (10.8), HCAR3 (6.7), DRD2 (6.3)	NR1H4 (−17.3), RXRG (−15.7), CNR1 (−11.1), HTR3C (−11.0), P2RY4 (−10.4)
TCGA-ESCA (Esophageal Carcinoma)	HTR2C (22.7), S1PR5 (6.2), P2RY6 (3.8), GPR84 (3.7), HTR1D (3.4)	HTR1E (−203.1), HRH2 (−14.2), CHRM2 (−12.3), P2RY14 (−12.0), RXRG (−8.3)
TCGA-GBM (Glioblastoma Multiforme)	AR (5.5), S1PR3 (5.5), P2RY8 (5.4), ADORA3 (4.8), TRPV4 (4.7)	HTR1A (−186.3), HTR3B (−95.4), HTR2C (−64.8), GABRB2 (−52.8), DRD1 (−51.9)
TCGA-HNSC (Head and Neck Squamous Cell Carcinoma)	GABRG2 (23.7), HTR2C (19.6), HTR1D (11.9), HTR7 (5.8), CHRM5 (4.1)	CHRM1 (−20.4), HTR3B (−18.0), ADRA1A (−10.5), PPARGC1A (−7.2), DRD2 (−5.0)
TCGA-KICH (Kidney Chromophobe)	CHRM1 (32.0), DRD2 (22.9), HTR3A (11.2), HCAR3 (8.7), HCAR2 (6.0)	NR1H4 (−16.2), DRD1 (−13.9), ADRA2C (−13.6), GPBAR1 (−9.8), HTR2B (−9.3)
TCGA-KIRC (Kidney Renal Clear Cell Carcinoma)	HTR6 (56.9), HRH2 (11.4), ADORA3 (8.1), FFAR4 (7.6), FFAR3 (6.2)	HTR3B (−38.9), ADRB1 (−9.1), HCAR1 (−6.4), HTR3C (−4.4), GABRB2 (−4.4)
TCGA-KIRP (Kidney Renal Papillary Cell Carcinoma)	TRPM8 (38.6), HTR3A (27.4), ADORA3 (10.3), FFAR4 (9.8), CNR1 (9.3)	HTR3B (−36.8), DRD1 (−9.8), RXRG (−7.6), ADRB1 (−7.0), GPBAR1 (−6.6)
TCGA-LIHC (Liver Hepatocellular Carcinoma)	GABRG2 (35.6), HTR2C (20.1), HTR1D (11.3), GPR35 (10.0), HTR3A (8.8)	CHRM2 (−22.5), ADRA1A (−8.8), ESR1 (−5.8), P2RY12 (−5.1), ADRA2B (−4.7)
TCGA-LUAD (Lung Adenocarcinoma)	TRPM8 (107.7), HTR3A (63.4), GABRG2 (14.9), HTR2C (9.5), P2RY6 (7.8)	HTR3C (−43.4), CHRM1 (−28.4), ADRA1A (−15.6), CHRM2 (−14.9), ADRB1 (−10.3)
TCGA-LUSC (Lung Squamous Cell Carcinoma)	HTR2C (203.8), GABRG2 (150.4), TRPM8 (11.0), HTR3A (10.1), HTR1E (9.6)	CHRM2 (−29.6), ADRA1A (−21.4), CHRM1 (−19.3), HTR3C (−17.0), FFAR4 (−12.9)
TCGA-PAAD (Pancreatic Adenocarcinoma)	HTR1D (6.1)	CNR2 (−23.2), ADRA1A (−11.9)
TCGA-PCPG (Pheochromocytoma and Paraganglioma)	HTR1E (91.6), HRH3 (70.5), DRD2 (66.0), HTR6 (54.1), ADORA1 (45.0)	LPAR3 (−55.0), NR1H4 (−37.7), P2RY2 (−13.8), VDR (−13.4), ESR2 (−7.4)
TCGA-PRAD (Prostate Adenocarcinoma)	FFAR2 (3.5), HTR4 (3.1), TRPM8 (2.4), AHR (2.4), HTR3A (2.3)	ADRB3 (−5.8), CHRM2 (−4.5), HCAR2 (−4.2), HTR1E (−4.1), HCAR3 (−4.0)
TCGA-READ (Rectum Adenocarcinoma)	HCAR1 (15.8), HTR1D (9.9), DRD2 (9.1), HCAR3 (7.1), ADRA2C (5.7)	CHRM2 (−134.0), CNR1 (−35.4), GABRG2 (−25.0), GPR119 (−18.8), RXRG (−16.2)
TCGA-SARC (Sarcoma)	DRD2 (102.7)	
TCGA-STAD (Stomach Adenocarcinoma)	HTR2C (24.1), TRPM8 (4.4), GPR84 (4.1), DRD1 (3.4), P2RY6 (2.8)	HTR1E (−16.4), RXRG (−9.9), CHRM2 (−6.5), HTR4 (−6.0), ADRB3 (−5.8)
TCGA-THCA (Thyroid Carcinoma)	GABRB2 (159.5), RXRG (47.2), GABRA1 (14.8), LPAR5 (11.0), ADRB3 (9.4)	HTR1E (−6.3), HTR3C (−4.5), PPARGC1A (−4.0), CNR2 (−3.8), HTR2A (−2.8)
TCGA-THYM (Thymoma)		RXRG (−120.3), PPARG (−11.6), GPBAR1 (−6.2)
TCGA-UCEC (Uterine Corpus Endometrial Carcinoma)	HTR3A (24.2), HRH3 (15.6), HTR2C (8.4), HTR6 (8.3), TRPM8 (6.5)	ADRB3 (−34.9), HTR1E (−19.1), HTR2A (−16.4), ADRA1D (−11.1), HTR2B (−9.3)

## Data Availability

All pre-processed TCGA data are available publicly available online through the recount3 repository at https://rna.recount.bio/ (accessed on 11 December 2024). CCLE data are available Cancer Cell Line Encyclopedia (CCLE 2019) https://sites.broadinstitute.org/ccle/datasets (accessed on 11 December 2024). All relevant scripts used for the analyses of data and the production of the figures presented in this work can be found on GitHub (https://github.com/ndovro/MMR) (accessed on 11 December 2024).

## Supplementary Materials

The following supporting information can be downloaded at: https://www.mdpi.com/article/10.3390/cancers16244178/s1, Figure S1: Cell Lines with peak expression of each MMR; Table S1: Summarized dysregulation of all MMRs across cancer types; Table S2: Spearman correlation *p*-values between cancer-types based on their MMR expression-profiles; Table S3: Aggregated list of cancer hallmark genes; Table S4: Pairwise Spearman correlation of the expression of all cancer hallmark genes and MMRs across all cancers.

## Abbreviations

**Abbreviation**	**Definition**
CCLE	Cancer Cell Line Encyclopedia
CHG	Cancer hallmark gene
CHP	Cancer hallmark pathway
GPCR	G-protein coupled receptors
HDAC	Histone deacetylase
MMR	Microbial metabolite receptor
SCFA	Short-chain fatty acid
TCGA	The Cancer Genome Atlas
TPM	Transcripts per million
