# Supplementary figures and images for: Pan-Cancer Insights: A Study of Microbial Metabolite Receptors in Malignancy Dynamics
Source: Cancers (Basel). 2024 Dec 15;16(24):4178. doi: 10.3390/cancers16244178 (PMC11674037; doi:10.3390/cancers16244178)

Highest Mean Normalized TPM by Site for Each Gene (log2)

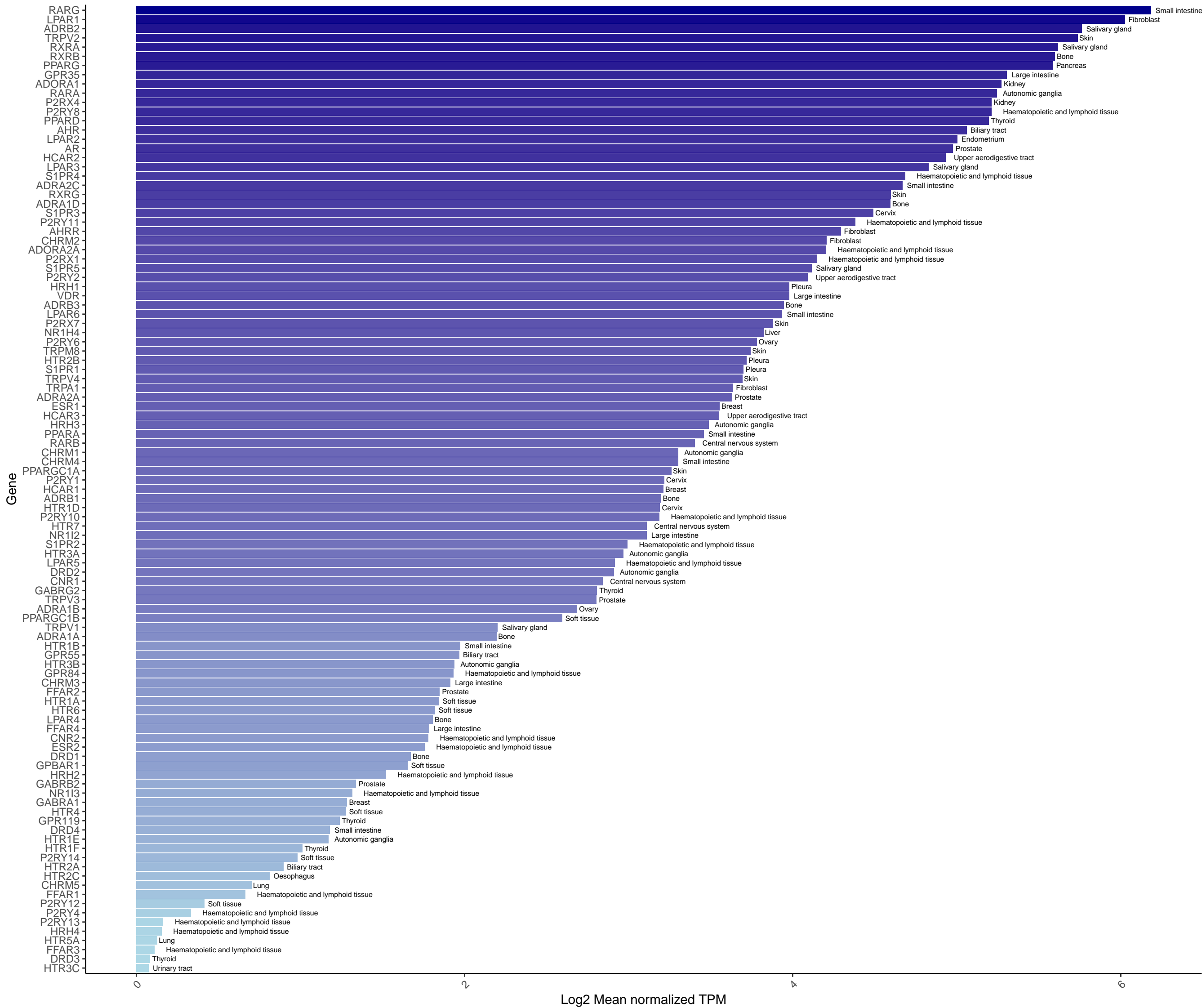

Supplement: Supplementary file 1 [file cancers-16-04178-s001.zip › Supplementary_figure S1.pdf]
